# The Prognostic Significance and Gene Expression Characteristics of Gastric Signet-Ring Cell Carcinoma: A Study Based on the SEER and TCGA Databases

**DOI:** 10.3389/fsurg.2022.819018

**Published:** 2022-03-17

**Authors:** Junren Ma, Yan Meng, Xin Zhou, Limei Guo, Wei Fu

**Affiliations:** ^1^Department of General Surgery, Peking University Third Hospital, Beijing, China; ^2^Peking University Third Hospital Cancer Center, Beijing, China; ^3^Department of Pathology, Peking University Third Hospital, Beijing, China

**Keywords:** signet-ring cell carcinoma, intestinal-type gastric cancer, clinical stages, prognostic factor, genomic profile

## Abstract

**Purpose:**

This study is based on the Surveillance, Epidemiology, and End Results (SEER) program to explore the prognostic differences between signet-ring cell carcinoma (SRC) and intestinal-type gastric carcinoma (ITGC). This study is also based on gene sequencing data from The Cancer Genome Atlas (TCGA) to identify unique genetic contributions to the prognostic differences between the two subtypes of gastric cancer.

**Patients and Methods:**

The clinical data were based on the SEER database from 2004 to 2015. Kaplan–Meier (KM) curves were used to compare 5-year overall survival (OS), and Cox regression was used for univariate and multivariate analyses. Gene expression profiles were obtained from TCGA database, and differentially expressed genes (DEGs) were screened. Functional enrichment analysis, protein interaction and survival analysis will be further carried out. Genes of interest were verified by the Human Protein Atlas, immunohistochemistry, and encyclopedia of Cancer Cell Lines (CCLE). The relationship between genes of interest and immune cell infiltration was also analyzed by Tumor Immune Estimation Resource (TIMER).

**Results:**

Compared with ITGC patients, SRC patients were more likely to be female, tended to be younger, and have a greater tumor distribution in the middle and lower stomach (*p* < 0.01). SRCs showed a significantly better prognosis than ITGCs (*p* < 0.01) in early gastric cancer (EGC), while the prognosis of SRCs was significantly worse than ITGCs (*p* < 0.05) in advanced gastric cancer (AGC). A total of 256 DEGs were screened in SRCs compared to ITGCs, and the enrichment analysis and protein interactions revealed that differential genes were mainly related to extracellular matrix organization. Thrombospondin1 (THBS1) and serpin peptidase inhibitor, clade E, member 1 (SERPINE1) are significantly differentially expressed between SRC and ITGC, which has been preliminarily verified by immunohistochemistry and open-source databases. THBS1 and SERPINE1 are also associated with multiple immune cell infiltrates in gastric cancer.

**Conclusions:**

There were significant differences in the clinicopathological features and prognosis between SRC and ITGC. These results suggest that SRC and ITGC may be two distinct types of tumors with different pathogeneses. We found many codifferentially expressed genes and important pathways between SRC and ITGC. THBS1 and SERPINE1 were significantly differentially expressed in the two types of gastric cancer, and may have potentially important functions.

## Introduction

With the advancements to the standard treatment of *Helicobacter pylori* (HP), the overall incidence of gastric cancer is declining (1). However, the incidence of signet-ring cell carcinoma (SRC) is increasing each year (2). SRC is a subtype of gastric cancer with a large amount of mucus (3) and is generally considered to have a poor prognosis (4, 5). In recent years, some studies in Asia have shown that the prognosis of SRC is closely related to clinical stage (6–13). SRC has a good prognosis in the early stage and a relatively worse prognosis in the advanced stage. Only a few western studies have analyzed SRC vs. non-signet ring cell carcinoma (NSRC), but the preliminary conclusion is not consistent with that of Eastern countries (14, 15). Intra-tumor heterogeneity may lead to unexpected bias; hence, the need to compare SRC with intestinal-type gastric carcinoma (ITGC). Meanwhile, the expression characteristics of SRC at the gene level have not been specifically and clearly explained. The purpose of this study was to investigate the prognostic significance of SRC and ITGC based on the Surveillance, Epidemiology, and End Results (SEER) database and the gene expression characteristics of both types of cancer based on The Cancer Genome Atlas (TCGA) database.

## Methods

### Clinical Data

**Figure 1:**
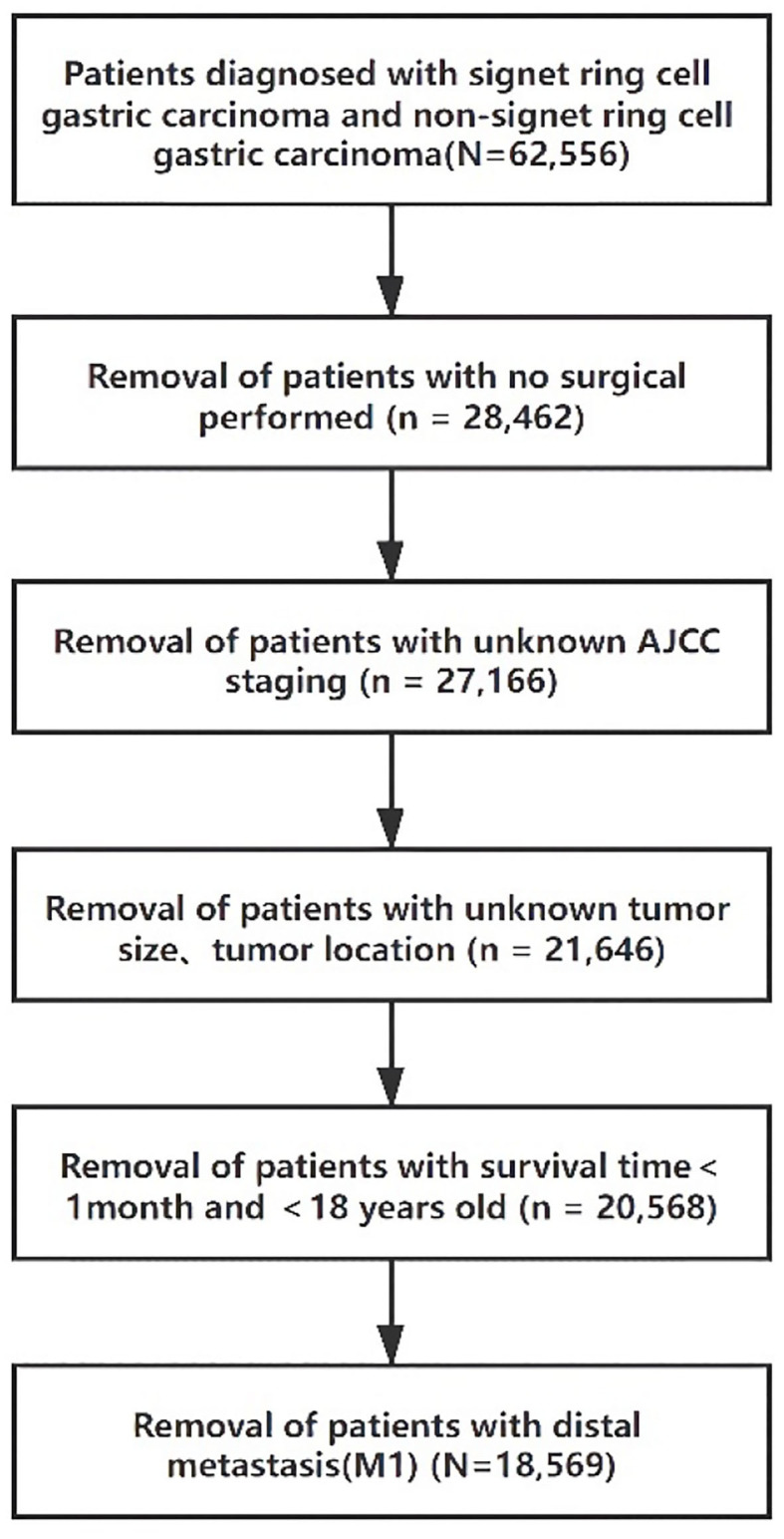
Flow diagram of selected cases in the surveillance, epidemiology, and end results database.

Clinical data were obtained from 18 SEER registries, and records from 2004 to 2007 were analyzed for this study. Data used for analysis included age, sex, race, tumor location, surgical treatment, pathological stage, lymph node metastasis status, and survival status. SRC is defined as adenocarcinoma in which more than 50% of the tumor consists of isolated or small groups of malignant cells containing intracytoplasmic mucin (3). Early gastric cancer (EGC) is defined as a tumor limited to the mucosa or submucosa, regardless of lymph nodal status (16). Advanced gastric cancer (AGC) is defined as tumor invasion beyond the submucosa. The International Classification of Diseases (ICD) code 8490/3 was used to identify SRC patients, while the codes 8140/3, 8144/3, 8210/3, 8211/3, 8260/3, 8261/3, 8262/3, and 8283/3 were used for ITGC patients. For the 57,200 patients with SRC and ITGC, the exclusion criteria were as follows: unknown surgery status (*n* = 32,341), unknown staging (*n* = 908), unknown differential (*n* = 1,481), race, tumor size, unknown tumor location (*n* = 4,250), survival time <1 month (*n* = 1,031), <18 years old (*n* = 4), M1 (*n* = 1,662) (Figure 1). The final analysis patients (*N* = 16,123) were divided into three groups according to the WHO histological type: well-to-moderately differentiated adenocarcinoma (WMD, *n* = 6,107), poorly differentiated adenocarcinoma (PD, *n* = 6,518), and SRC (*n* = 3,498).

### RNA Sequencing Data

**Figure 2:**
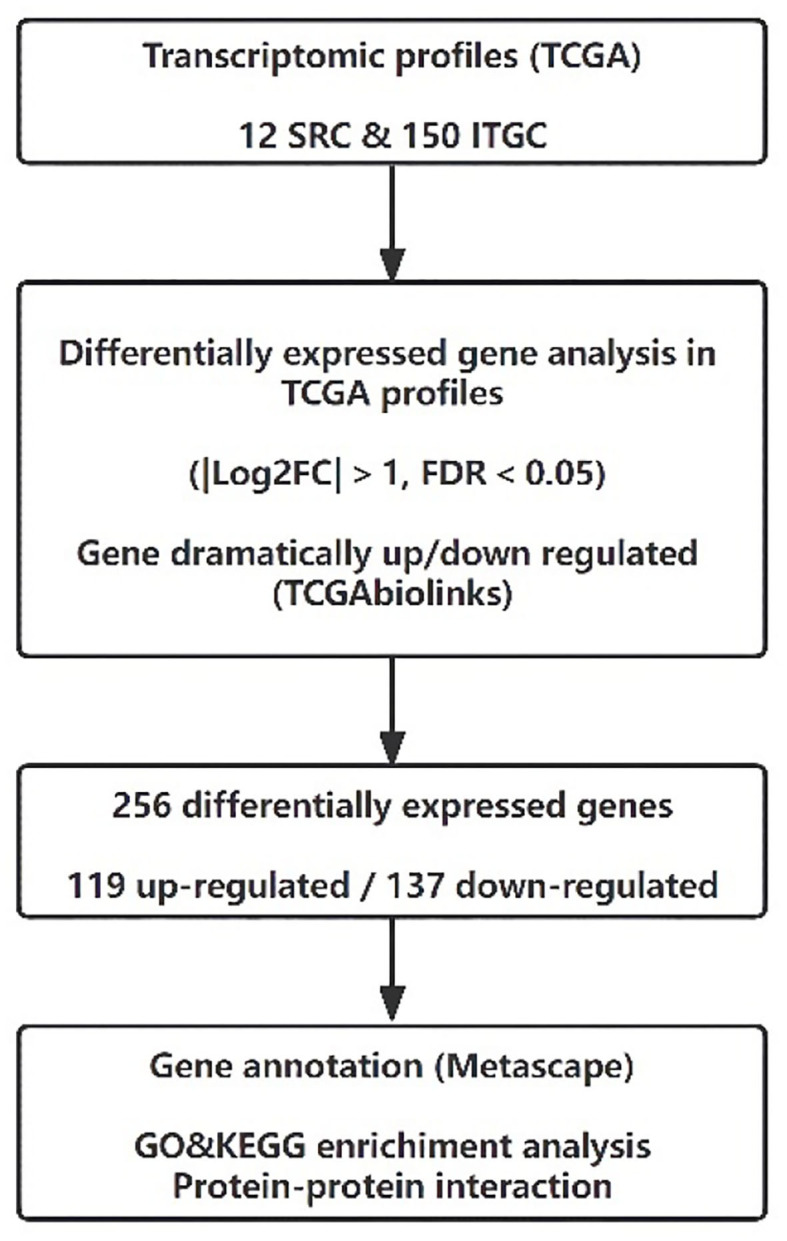
Flow diagram of transcriptomic profiles in The Cancer Genome Atlas database.

The RNA sequencing data of SRC and ITGC patients were obtained from the TCGA database. The inclusion criteria of gastric cancer samples were as follows: (i) gene expression profiling of SRC and ITGC were available in the dataset; (ii) the ICD code 8490/3 was used to identify SRC patients, while the codes 8144/3, 8211/3, and 8260/3 were used for ITGC patients. Finally, 12 SRC patients and 150 ITGC patients were enrolled in this study. Our workflow for the bioinformatics analysis of TCGA databases is illustrated in Figure 2.

### Genome Sequencing Data Analysis

The RNA sequencing results of enrolled patients were obtained from TCGA data portal (https://tcga-data.nci.nih.gov/tcga/). They were normalized and processed with TCGAbiolinks of R software (17). The TCGAbiolinks principle of differential analysis was first used to convert the count matrix into an edgeR object (18), and then it assigned the same discrete estimate to each gene. Then, a pairwise test was used to identify the differential expression patterns between SRC and ITGC. Finally, the error detection rate (FDR) correction was used to obtain the output and identify differentially expressed genes (DEGs). The parameters set for the differential expression analysis were FDR < 0.05 with |Log_2_FC| > 1.

### Analysis and Validation of Interest Genes

Gene Ontology (GO) and Kyoto Encyclopedia of Genes and Genomes (KEGG) pathway enrichment analyses and protein–protein interaction (PPI) analysis were then performed using Metascape (http://metascape.org) (19). Kaplan–Meier (KM) plots of the genes of interest were constructed. The overall survival (OS) was analyzed, and the log-rank test was performed. Pearson's test was used for pairwise gene expression correlation analysis of the genes of interest. A *p* < 0.05 was considered to be significant.

Immunohistochemical methods were used to verify the genes of interest in postoperative pathological tissues of gastric cancer in our hospital. The pathological tissues were obtained from postoperative specimens from Peking University Third Hospital and included SRC and ITGC tissues. These samples were evaluated by an independent pathologist. Tissues (5 mm thick) were deparaffinized and treated with 3% H_2_O_2_-CH_3_OH for 15 min to block endogenous peroxidase. Samples were submerged in a pH 6.0 or 9.0 buffer in a pressure cooker for antigen retrieval and then incubated at 37°C for 2 h with antibodies against thrombospondin 1 (Abcam, ab1823, 1:50) and PAI1 (Abcam, ab125687, 1:50). After washing with phosphate-buffered saline (PBS), the sections were incubated with horseradish peroxidase (HRP)-conjugated IgG (ZSGB—Bio, PV-6000) at room temperature for 30 min and then stained with a 3,3N-diaminobenzidine tetrahydrochloride (DAB) detection system kit (ZSGB-Bio, ZLI-9018). Protein expression and localization were detected under light microscopy and analyzed by Nikon Diagram Program (NDP) view (version 2.6.8).

We used the Human Protein Atlas and Encyclopedia of Cancer Cell Lines (CCLE) databases to verify the expression of genes of interest in pathological tumor tissues and tumor cell lines. We also use Tumor Immune Estimation Resource (TIMER) (http://cistrome.org/TIMER) (20) to further explore the clinical effects of differential genes and different immune invasions infiltrates.

### Statistical Analysis

Continuous variables and categorical variables were compared by *t*-test and chi-square analysis, respectively. The KM method was used to calculate the survival rate, and then the survival curves were compared by the log-rank test. Univariate and multivariate analyses were performed by the Cox regression risk model. All data analyses were performed by SPSS version 24.0.

This study conforms to the Strengthening the reporting of cohort studies in surgery (STROCSS) criteria (21). Because all the original data come from open-source databases, ethical review is unnecessary.

## Results

### Clinicopathological Characteristics

The clinicopathologic characteristics of patients with EGC and AGC are shown in Tables 1, 2, respectively. Of the 16,123 gastric cancer patients, 4,271 patients (26.5%) had EGC, and 11,852 patients (73.5%) had AGC. There was a statistically significant difference (*p* < 0.01) in histological type between the EGC patients and the AGC patients.

**Table 1:** Comparison of clinicopathological characteristics in early gastric cancer.

	**SRC** **75,417.7%**	**WMD** **240,056.2%**	***p1*** **(SRC&WMD)**	**PD** **111,726.1%**	***p2*** **(SRC&PD)**
**Age (Mean ± SD)**	63.4 ± 13.5	70.6 ± 10.6	<0.01	68.9 ± 11.2	<0.01
≤ 60 years old	29,038.5%	42,817.8%		24,421.8%	
>60 years old	46,461.5%	197,282.2%		87,378.2%	
**Gender**			<0.01		<0.01
Male	35,947.6%	159,766.5%		70,763.3%	
Female	39,552.4%	80,333.5%		41,036.7%	
**Size (Mean ± SD)**	22.8 ± 19.4	22.3 ± 18.7	0.541	26.3 ± 19.8	<0.01
≤ 2 cm	43,858.1%	139,658.2%		54,849.1%	
>2 cm	31,641.9%	100,441.8%		56,950.9%	
**Race**			0.412		0.423
White	49,365.4%	162,567.7%	0.239	70,763.3%	0.349
Black	8,911.8%	25,210.5	0.324	14,212.7%	0.543
American Indian	60.8%	150.6%	0.612	70.6%	0.657
Asian/Pacific Islander	16,622%	50,821.2%	0.623	26,123.4%	0.489
**Location**			<0.01		<0.01
Upper	12,416.4%	95,539.8%	<0.01	38,334.3%	<0.01
Middle	29,238.7%	56,723.6%	<0.01	32,328.9%	<0.01
Lower	30,240.1%	79,633.2%	<0.01	36,232.4%	<0.01
**Whole**	364.8%	823.4%	0.093	494.4%	0.671
pN			<0.01		<0.01
N–	60,480.1%	209,687.3%	<0.01	83,474.7%	<0.01
N+	15,019.9%	30,412.7%	<0.01	28,325.3%	<0.01

*SRC, signet ring cell carcinoma; WMD, well-to-moderately differentiated adenocarcinoma; PD, poorly differentiated adenocarcinoma; SD, standard deviation*.

**Table 2:** Comparison of clinicopathological characteristics in advanced gastric cancer.

	**SRC** **2744,23.2%**	**WMD** **3707,31.2%**	***P1*** **(SRC&WMD)**	**PD** **5401,45.6%**	***P2*** **(SRC&PD)**
**Age (Mean ± SD)**	62.2 ± 13.5	68.6 ± 11.5	<0.01	67.8 ± 12.4	<0.01
≤ 60 years old	120,343.8%	87,523.6%		147,227.3%	
>60 years old	154,156.2%	283,276.4%		392,972.7%	
**Gender**			<0.01		<0.01
Male	148,354%	262,370.8%		359,966.6%	
Female	126,146%	108,429.2%		180,233.4%	
**Size (Mean ± SD)**	56.2 ± 36.3	47.0 ± 24.6	<0.01	51.8 ± 27.1	<0.01
≤ 5 cm	144,852.8%	237,063.9%		312,057.8%	
>5 cm	129,647.1%	133,736.1%		228,142.2%	
**Race**			0.062		0.486
White	1857,67.7%	2541,68.5%	0.457	3696,68.4%	0.488
Black	350,12.8%	521,14.1%	0.127	686,12.7%	0.946
American Indian	23,0.8%	37,1%	0.462	29,0.5%	0.137
Asian/Pacific Islander	514,18.7%	608,16.4%	0.016	990,18.3%	0.654
**Location**			<0.01		<0.01
Upper	56,720.7%	153,241.3%	<0.01	196,336.3%	<0.01
Middle	87,631.9%	86,923.4%	<0.01	144,626.8%	<0.01
Lower	97,635.6%	111,430.1%	<0.01	162,130%	<0.01
Whole	32,511.8%	1,925.2%	<0.01	3,716.9%	<0.01
**pT**			<0.01		<0.01
T2	38,414%	95,725.8%	<0.01	89,416.6%	<0.01
T3	119,943.7%	195,452.7%	<0.01	283,152.4%	<0.01
T4	116,142.3%	79,621.5%	<0.01	167,631%	<0.01
**pN**			<0.01		<0.01
N0	68,825.1%	152,441.1%	<0.01	147,627.3%	0.036
N1	83,230.3%	140,137.8%	<0.01	207,638.4%	<0.01
N2	62,722.8%	52,914.3%	<0.01	113,921.1%	0.056
N3	59,721.8%	2,536.8%	<0.01	71,013.1%	<0.01

*SRC, signet ring cell carcinoma; WMD, well-to-moderately differentiated adenocarcinoma; PD, poorly differentiated adenocarcinoma; SD, standard deviation*.

In patients with EGC, SRC was more common in younger patients and female patients than WMD (age: *p* < 0.01; sex: *p* < 0.01) and PD (age: *p* < 0.01; sex: *p* < 0.01). There was no significant difference in tumor size between the SRC and the WMD, but tumor size was smaller in SRC than PD (*p* < 0.01). Furthermore, there were more middle and lower third tumor locations and less upper third locations in SRC (*p* < 0.01). The SRC patients had less lymph node metastasis (LNM) than PD (*p* < 0.01) patients and more LNM than WMD (*p* < 0.01) patients.

In patients with AGC, SRC was more common in younger, female patients, and the tumor size was larger than that of WMD (age: *p* < 0.01; sex: *p* < 0.01; size: *p* < 0.01) and PD (age: *p* < 0.01; sex: *p* < 0.01; size: *p* < 0.01). There were more Asian/Pacific islanders in the SRC group than in the WMD group (*p* = 0.016). SRCs were more frequently located in the middle and lower third of the stomach than WMDs (*p* < 0.01) and PDs (*p* < 0.01), and SRCs presented a more diffuse infiltration growth pattern (*p* < 0.01). In the tumor stage (T) and lymph node (N) stage, the proportion of SRC patients with stage T4 and N3 disease was higher than that of WMD (*p* < 0.01) and PD (*p* < 0.01) patients.

### Survival

**Figure 3:**
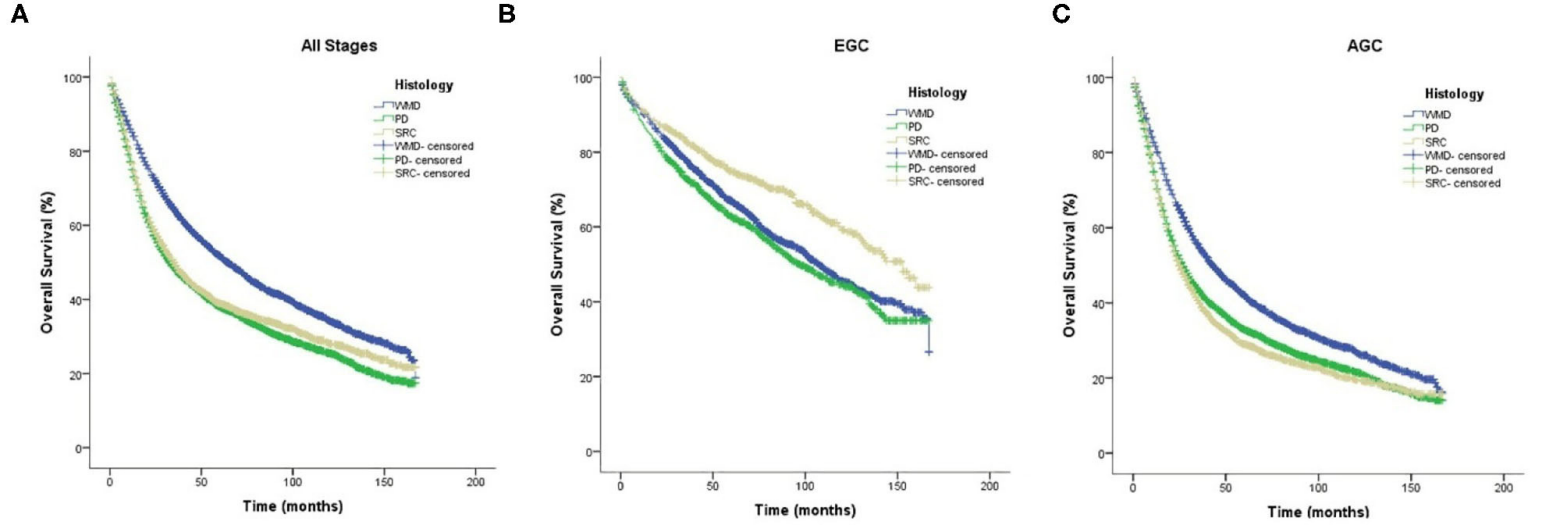
Kaplan–Meier survival curves comparing overall survival in signet-ring cell carcinoma and non-signet-ring cell carcinoma are shown for **(A)** all stages, **(B)** early gastric cancer, and **(C)** advanced gastric cancer.

The median follow-up was 35 months. The KM curves for different clinical stages are shown in Figure 3. In general, the OS of WMD was significantly better than that of SRC and PD (*p* < 0.01), and there was no significant difference between SRC and PD.

Notably, when the patients were divided into EGC and AGC by pathological stage, SRC showed a significantly better prognosis than both WMD and PD in EGC (*p* < 0.01). However, this result was reversed in AGC; that is, SRC demonstrated a significantly worse prognosis than WMD (*p* < 0.01) and PD (*p* = 0.041). Regardless of EGC or AGC, PD has a worse prognosis than WMD (*p* < 0.01).

### Mortality Predictors

We performed an unadjusted analysis of OS for EGC and AGC and performed a multivariate analysis using Cox's proportional hazard model after adjustments for sex, age, race, location, tumor size, and pathological stage.

In EGC, the univariate analysis showed that SRC was associated with a reduction in mortality compared to WMD (*HR*: 0.702; 95% *CI*: 0.611–0.807; *p* < 0.01) and PD (*HR*: 0.628; 95% *CI*: 0.539–0.730; *p* < 0.01), as shown in Table 3. Multivariate analysis showed that SRC was an independent protective factor for OS in EGC compared with WMD (*HR*: 0.859, 95% *CI*: 0.744–0.992, *p* = 0.039) and PD (*HR*: 0.767, 95% *CI*: 0.657–0.896, *p* < 0.01).

**Table 3:** Univariate and multivariate analyses of prognostic factors for overall survival in early gastric cancer.

	**Univariate**			**Multivariate**		
	**HR**	**95%CI**	***p***	**HR**	**95%CI**	***p***
**Gender (Male)**	1.226	1.111~1.353	<0.01	1.204	1.088~1.332	<0.01
**Age (≤60)**	0.379	0.328~0.437	<0.01	0.363	0.313~0.42	<0.01
**Race**						
White (vs. API)	1.547	1.363~1.756	<0.01	1.501	1.314~1.715	<0.01
**Location**						
Middle (vs. Upper)	0.794	0.705~0.895	<0.01	0.828	0.729~0.94	<0.01
Lower (vs. Upper)	0.817	0.730~0.913	<0.01	0.86	0.761~0.972	<0.01
**Histology**						
SRC (vs. ITGC)	0.677	0.592~0.775	<0.01	0.826	0.719~0.949	<0.01
SRC (vs. WMD)	0.702	0.611~0.807	<0.01	0.859	0.744~0.992	0.039
SRC (vs. PD)	0.628	0.539~0.730	<0.01	0.767	0.657~0.896	<0.01
**Tumor size (≤2 cm)**	0.793	0.722~0.87	<0.01	0.893	0.811~0.984	0.022
**pN (N–)**	0.693	0.571~0.715	<0.01	0.647	0.575~0.727	<0.01

*HR, hazard ratio; CI, Confidence interval; SRC, signet ring cell carcinoma; WMD, well-to-moderately differentiated adenocarcinoma; PD, poorly differentiated adenocarcinoma; ITGC, intestinal-type gastric carcinoma; API, Asian/Pacific Islander*.

Meanwhile, in AGC, the univariate analysis showed that SRC was associated with increased mortality compared to WMD (*HR*: 1.374; 95% *CI*: 1.294–1.458; *p* < 0.01) and PD (*HR*: 1.058; 95% *CI*: 1.002–1.116; *p* = 0.041), as shown in Table 4. Multivariate analysis showed that SRC was an independent risk predictor for OS in AGC compared with WMD (*HR*: 1.259, 95% *CI*: 1.182–1.182, *p* < 0.01) and PD (*HR*: 1.058, 95% *CI*: 1.001–1.119, *p* = 0.048).

**Table 4:** Univariate and multivariate analyses of prognostic factors for overall survival in advanced gastric cancer.

	**Univariate**			**Multivariate**		
	**HR**	**95% CI**	***p***	**HR**	**95% CI**	***p***
**Gender (Male)**	1.021	0.976~1.068	0.366	1.013	0.967~1.061	0.589
**Age (≤60)**	0.710	0.676~0.745	<0.01	0.620	0.590~0.652	<0.01
**Race**						
White (vs. API)	1.276	1.202~1.355	<0.01	1.292	1.216~1.374	<0.01
**Location**						
Middle (vs. Upper)	0.877	0.829~0.928	<0.01	0.822	0.775~0.871	<0.01
Lower (vs. Upper)	0.969	0.919~1.022	0.245	0.904	0.855~0.956	<0.01
**Histology**						
SRC (vs. ITGC)	1.178	1.12~1.238	<0.01	1.126	1.068~1.188	<0.01
SRC (vs. WMD)	1.374	1.294~1.458	<0.01	1.259	1.182~1.182	<0.01
SRC (vs. PD)	1.058	1.002~1.116	0.041	1.058	1.001~1.119	0.048
**Tumor size (≤5 cm)**	0.805	0.771~0.841	<0.01	0.965	0.922~1.01	0.122
**pT**						
T3 (vs. T2)	1.564	1.468~1.667	<0.01	1.343	1.258~1.433	<0.01
T4 (vs. T2)	2.274	2.127~2.430	<0.01	1.843	1.719~1.976	<0.01
**pN**						
N1 (vs. N0)	1.519	1.437~1.605	<0.01	1.397	1.321~1.478	<0.01
N2 (vs. N0)	1.915	1.796~2.041	<0.01	1.731	1.620~1.849	<0.01
N3 (vs. N0)	2.789	2.601~2.992	<0.01	2.442	2.269~2.628	<0.01

*HR, hazard ratio; CI, Confidence interval; SRC, signet ring cell carcinoma; WMD, well-to-moderately differentiated adenocarcinoma; PD, poorly differentiated adenocarcinoma; ITGC, intestinal-type gastric carcinoma; API, Asian/Pacific Islander*.

### Gene Expression Signatures of SRC and ITGC

**Figure 4:**
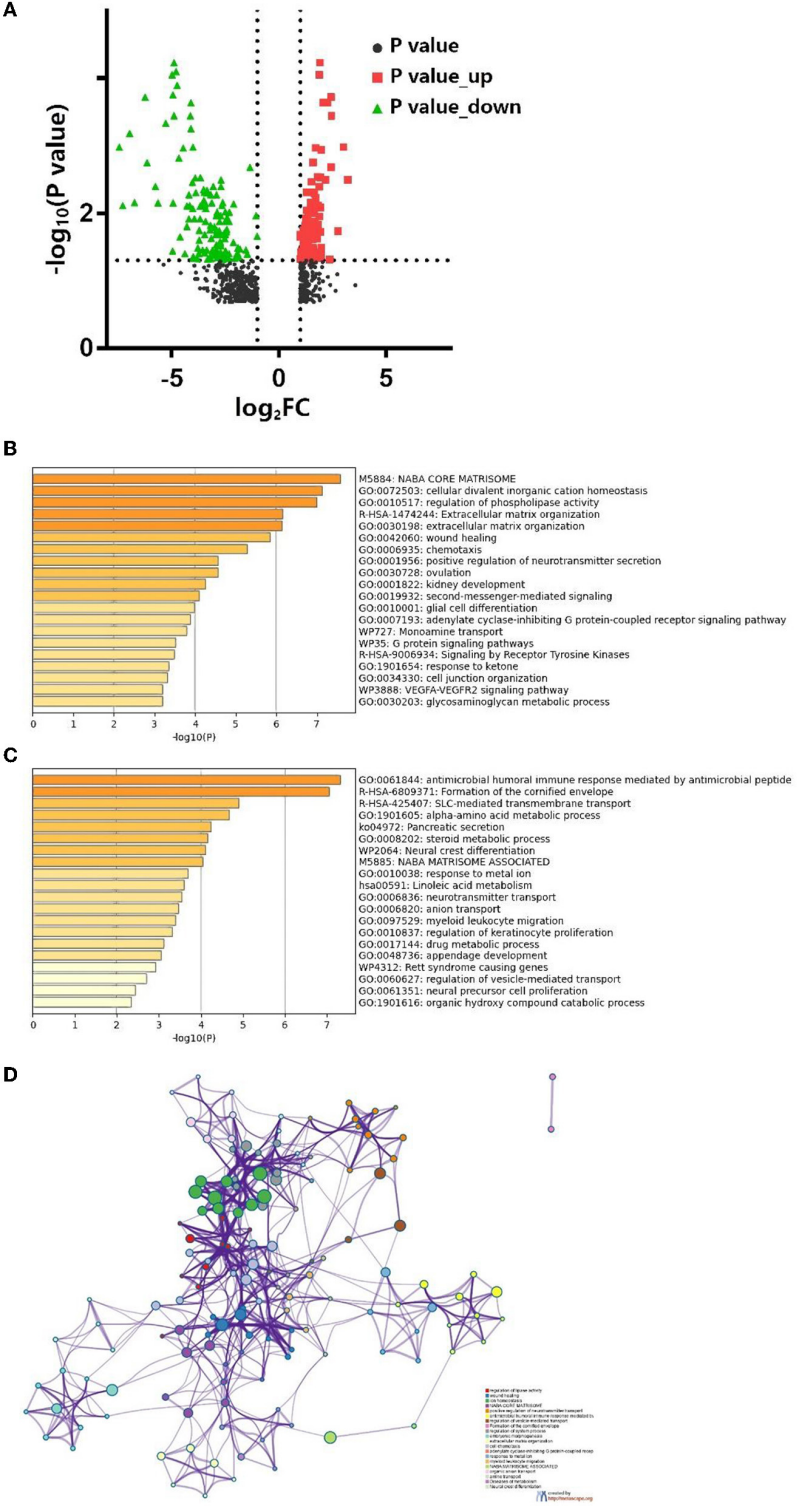
**(A)** Volcano map of differentially expressed genes. Bar graph of enriched terms across differentially expressed genes are shown for **(B)** up-regulate, **(D)** down-regulate, and **(C)** network of enriched terms colored by cluster ID, each node represents an enriched term. Nodes that share the same cluster ID are typically close to each other and terms with a similarity >0.3 are connected by edges.

We used the edgeR package (18) (|Log_2_FC| > 1, FDR <0.05) to identify DEGs. In total, 256 codifferentially expressed genes (119 upregulated and 137 downregulated) were found and are shown in volcano plots (Figure 4A). Further functional annotation was performed on these 256 genes to determine the meaningful biological processes in SRC. A bar graph of the enriched terms across the differentially expressed genes is shown in Figures 4B,D, and the network was visualized using Cytoscape (22) (Figure 4C).

**Figure 5:**
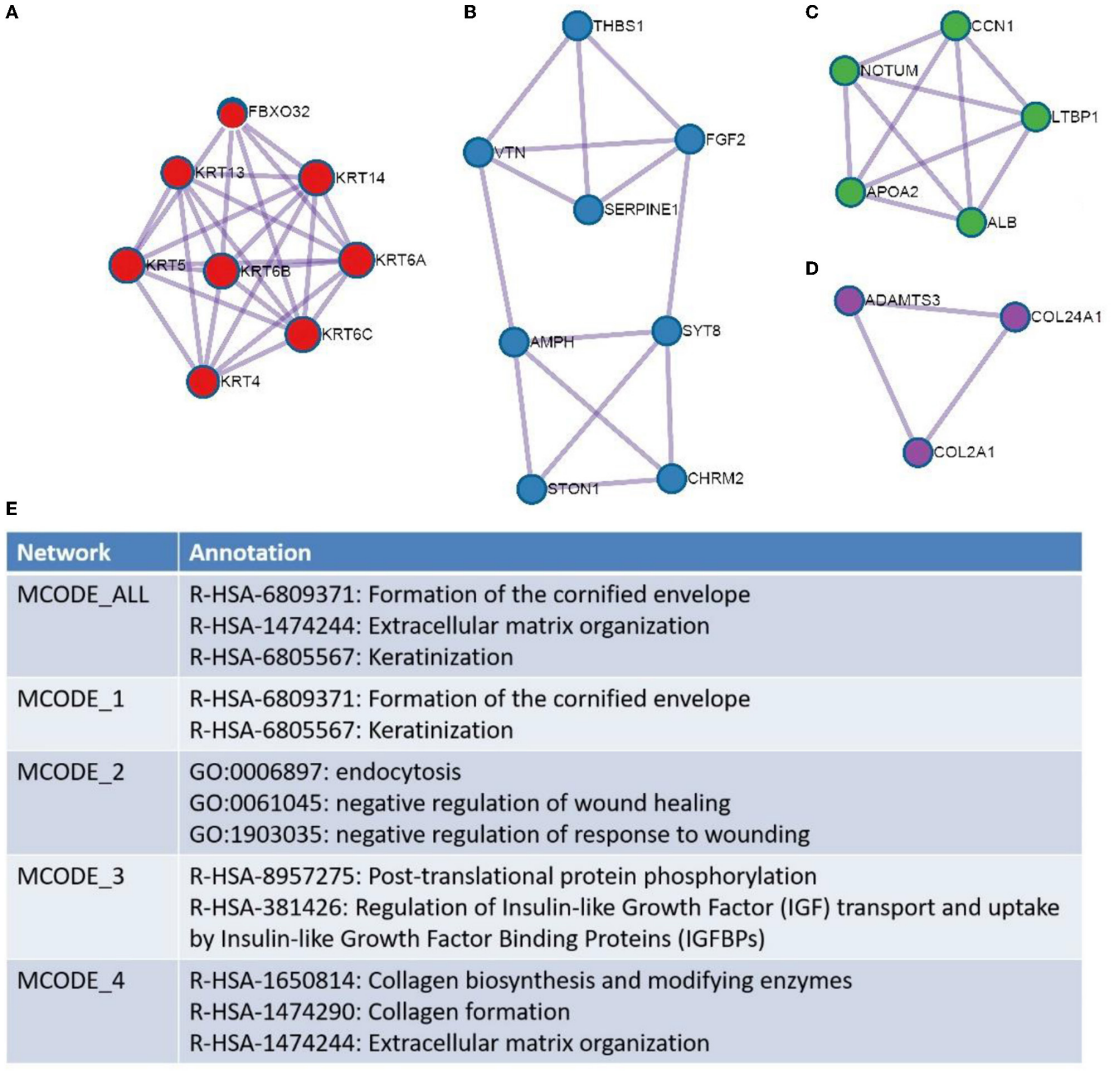
Protein–protein interaction network is shown for: **(A)** MCODE 1, **(B)** MCODE 2, **(C)** MCODE 3, and **(D)** MCODE 4. The resultant network contains the subset of proteins that form physical interactions with at least one other member in the list. If the network contains between 3 and 500 proteins, the Molecular Complex Detection (MCODE) algorithm has been applied to identify densely connected network components. Gene ontology enrichment analysis was applied to each MCODE network for **(E)**.

The results revealed that the biological processes primarily associated with the upregulated genes included the NABA core matrisome cellular divalent inorganic cation homeostasis, the regulation of phospholipase activity and extracellular matrix (ECM) organization. Furthermore, the downregulated genes were associated with the antimicrobial humoral immune response mediated by antimicrobial peptides and the formation of the cornified envelope. PPI enrichment analysis was performed with the following databases: Search tool for the retrival of interacting genes/proteins (STRING) (23), The Biological General Repository for Interaction Datasets (BioGrid) (24), OmniPath (25), and InWeb_IM (25). The molecular complex detection (MCODE) algorithm (26) was used to cluster the PPI network (Figures 5A–D) and GO enrichment analysis was applied to each MCODE network (Figure 5E). We found that the protein interactions were mainly related to the formation of the cornified envelope, ECM organization, and keratinization.

**Figure 6:**
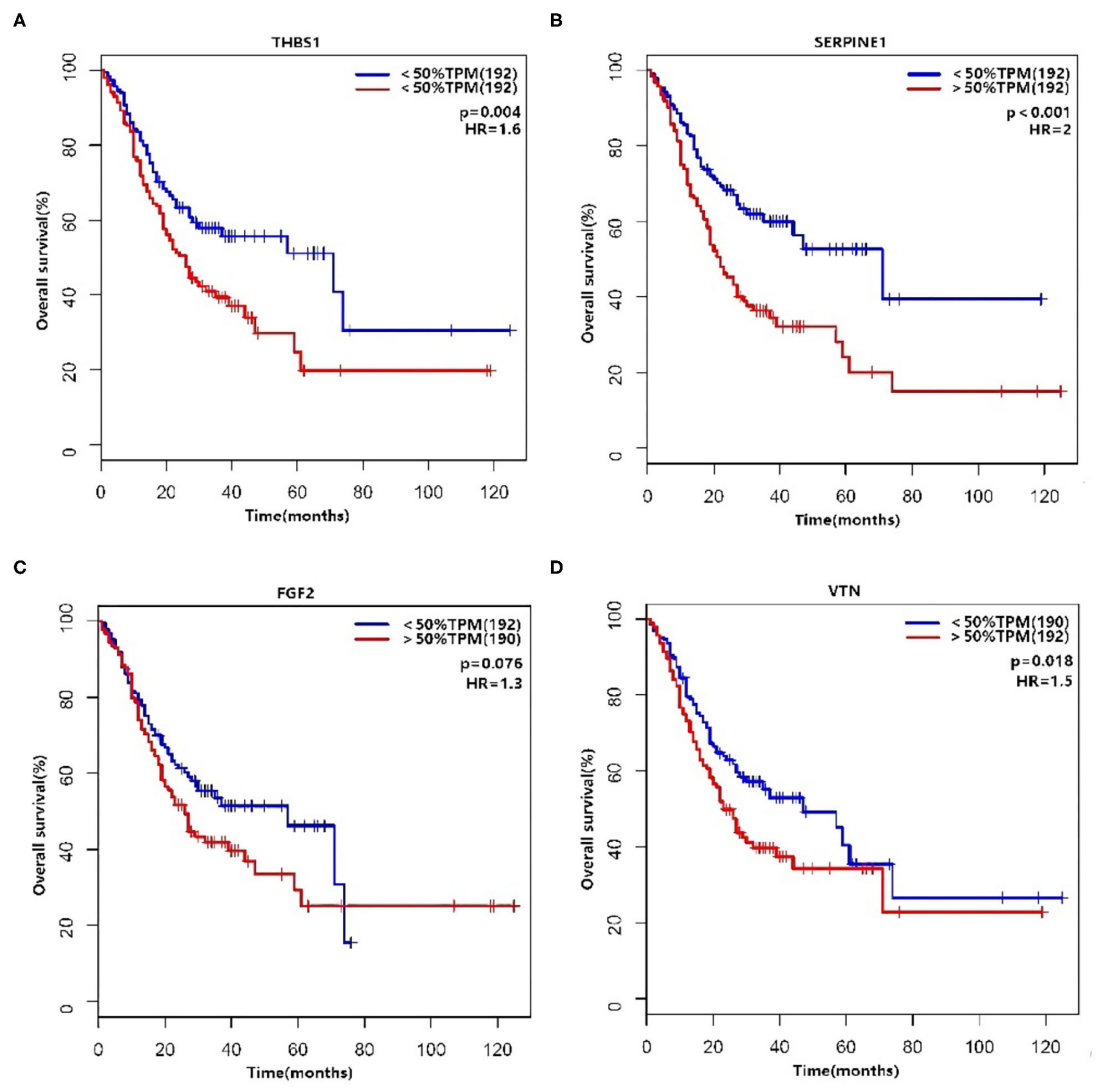
Survival analysis are shown for **(A)** THBS1, **(B)** SERPINE1, **(C)** FGF2, and **(D)** VTN.

**Figure 7:**
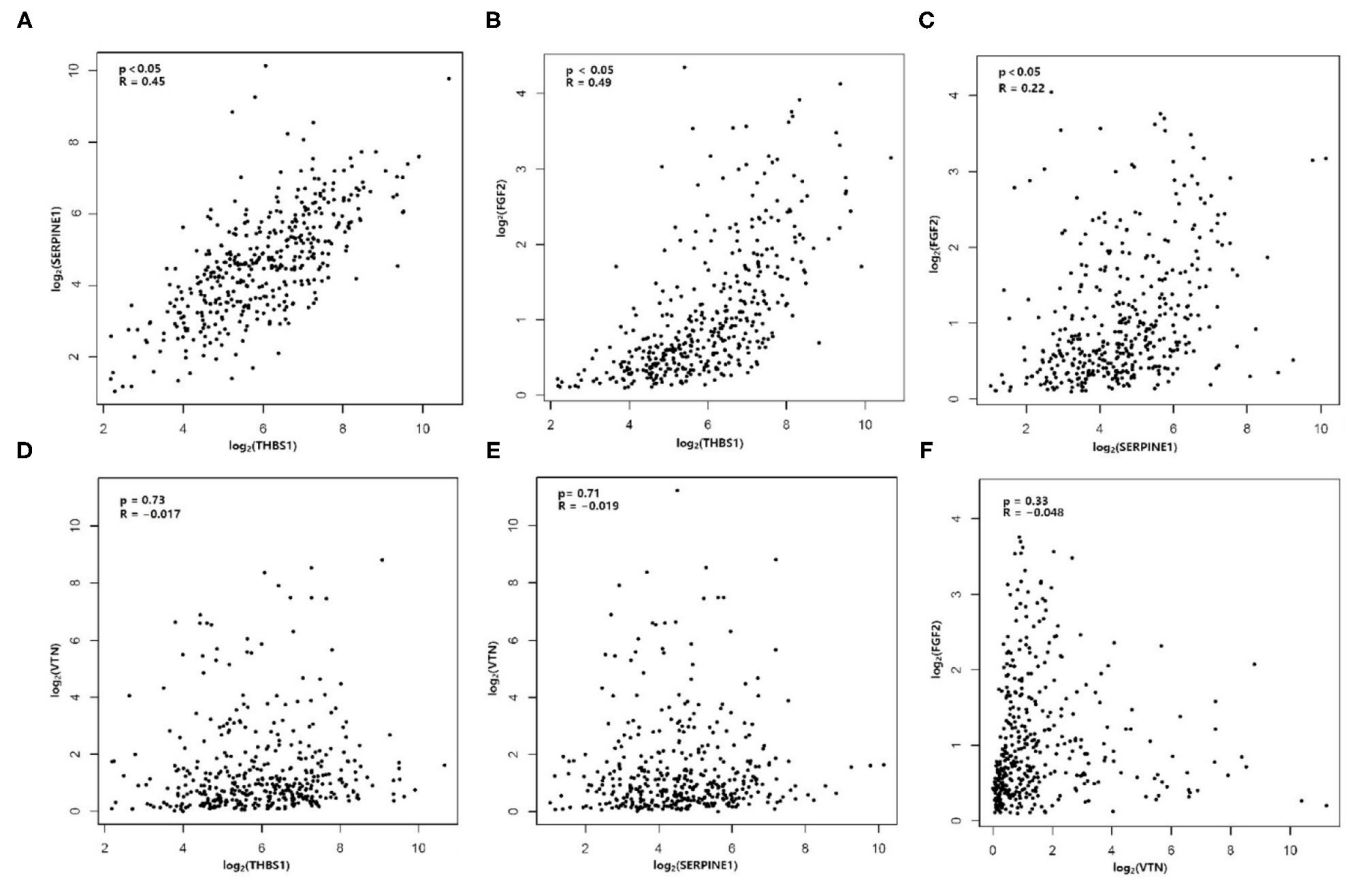
Correlation analysis is shown for **(A)** THBS1 and SERPINE1, **(B)** THBS1 and FGF2, **(C)** SERPINE1 and FGF2, **(D)** THBS1 and VTN, **(E)** SERPINE1 and VTN, and **(F)** VTN and FGF2.

There was an interaction between Thrombospondin1 (THBS1), serpin peptidase inhibitor, clade E, member 1 (SERPINE1), VTN, and FGF2. We used the median to classify high and low expression in terms of the expression level (TPM, transcripts per million). The KM method was used to calculate the survival rate between them (Figure 6), and then the survival curves were compared by the log-rank test. We found that THBS1, SERPINE1, and VTN were statistically related to OS (*p* < 0.05). The Pearson correlation coefficient was used for correlation analysis (Figure 7) based on the TPM of the DEGs and showed that THBS1, SERPINE1, and FGF2 were correlated.

### Validation of the Genes of Interest

**Figure 8:**
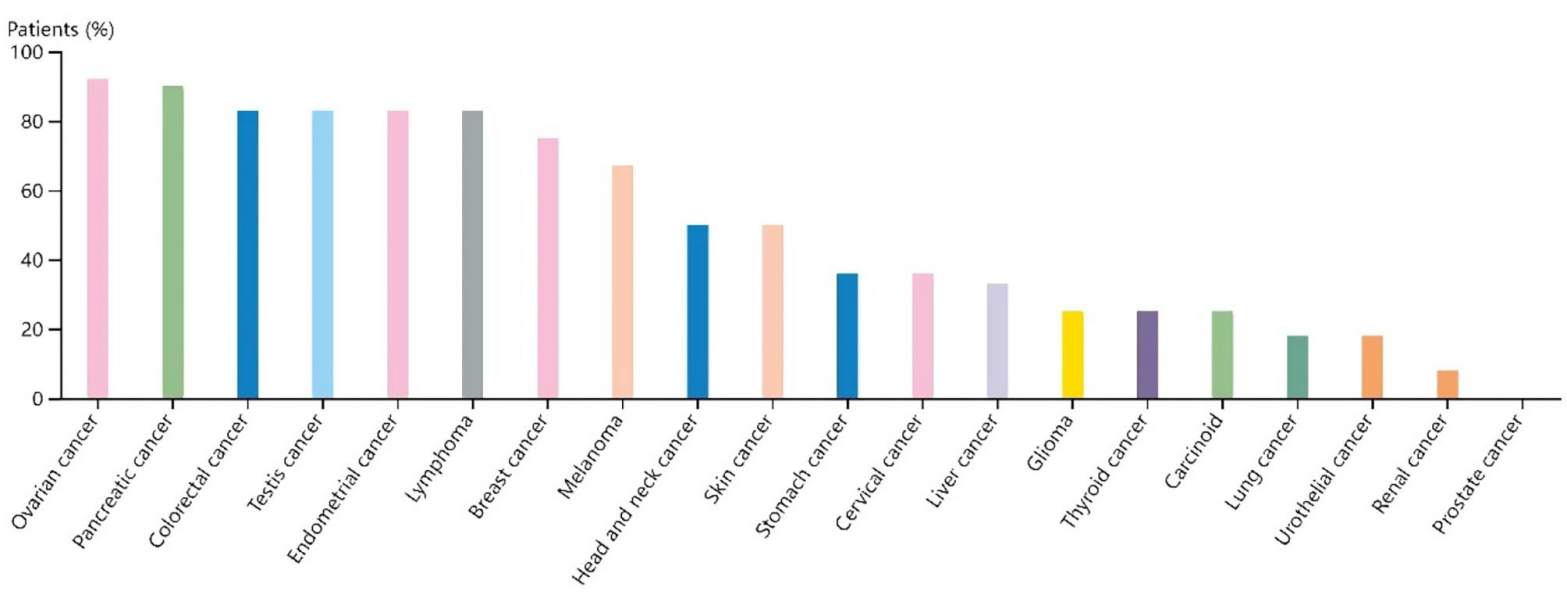
THBS1 showed moderate to strong membranous staining in malignant tumor cells, with occasional cytoplasmic staining. SERPINE1 was negatively expressed in all tumor tissues *via* The Human Protein Atlas.

Differential gene expression analysis suggested that THBS1 and SERPINE1 were significantly differentially expressed in the two types of gastric cancer, correlation analysis found a correlation between the two genes, and prognostic analysis suggested that THBS1 and SERPINE1 might have potential functions in SRC. We performed gene validation in the Human Protein Atlas and found that gastric cancers were partly positive for THBS1 (Figure 8).

**Figure 9:**
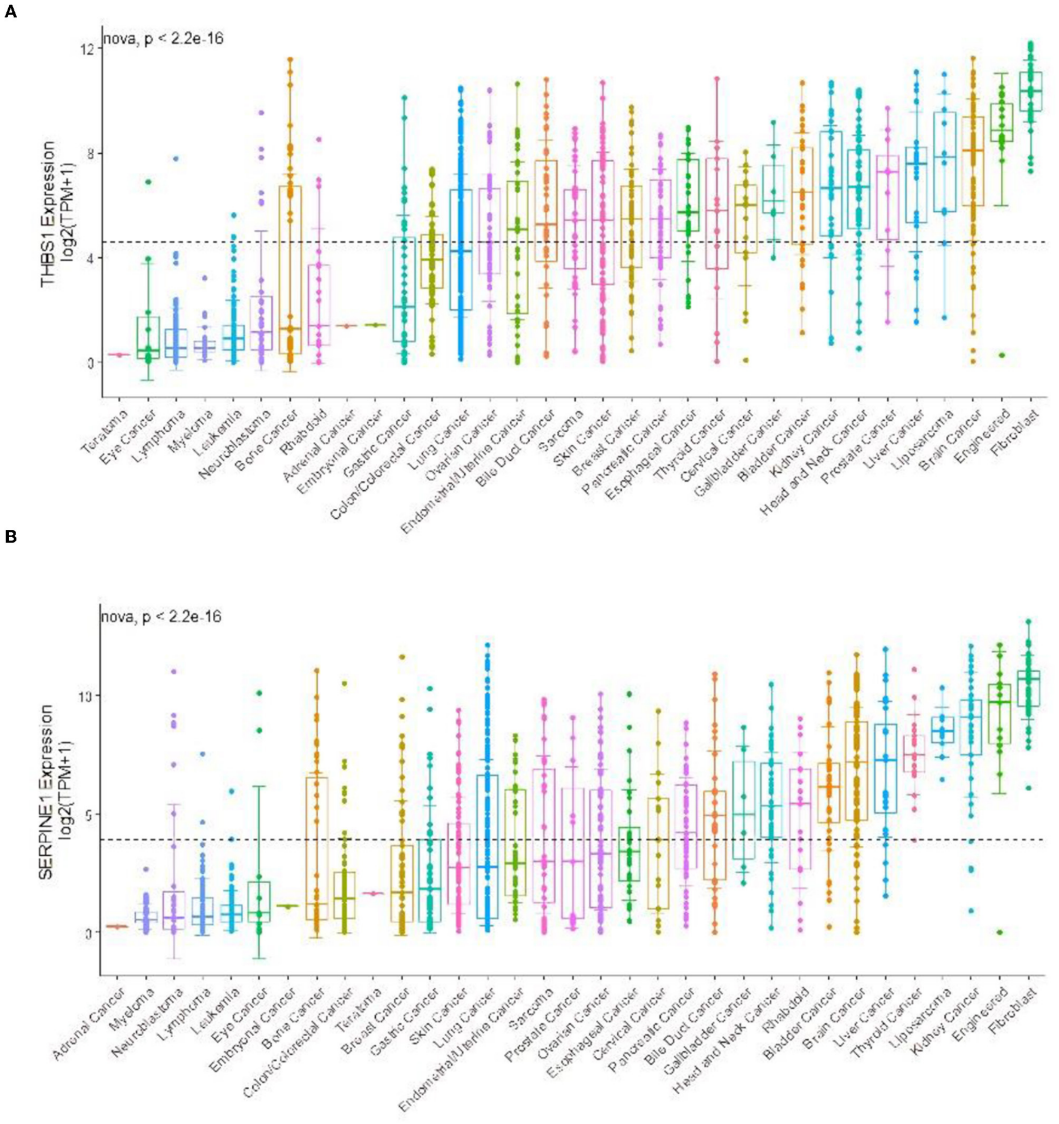
Gene expression in different cancer cell lines for **(A)** THBS1 and **(B)** SERPINE1. The black dotted lines represent the average expression of all tumors, and the horizontal lines in the middle of each boxplot represent the median expression of individual tumors.

**Figure 10:**
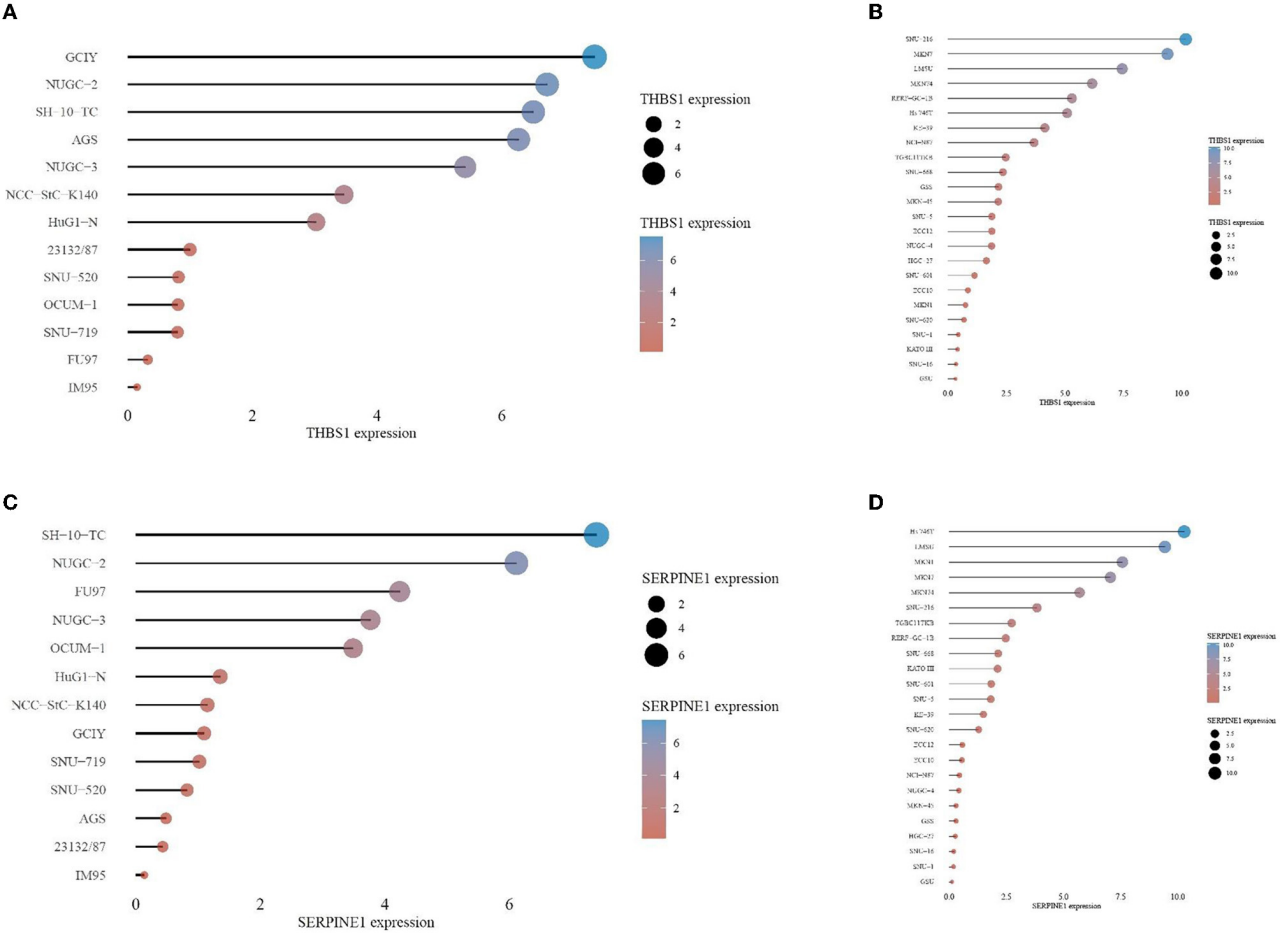
Gene expression levels in different gastric cancer cell lines for **(A)** THBS1 in primary cancer cell, **(B)** SERPINE1 in primary cancer cell, **(C)** THBS1 in metastasis cancer cell, and **(D)** SERPINE1 in metastasis cancer cell.

In the CCLE database, THBS1 was found to be highly expressed in many tumor cell lines but only moderately expressed in gastric cancer (Figure 9). Then, gastric cancer cell lines were analyzed, and SERPINE1 and THBS1 were found to be relatively higher in metastatic tumor cell lines (Figure 10).

**Figure 11:**
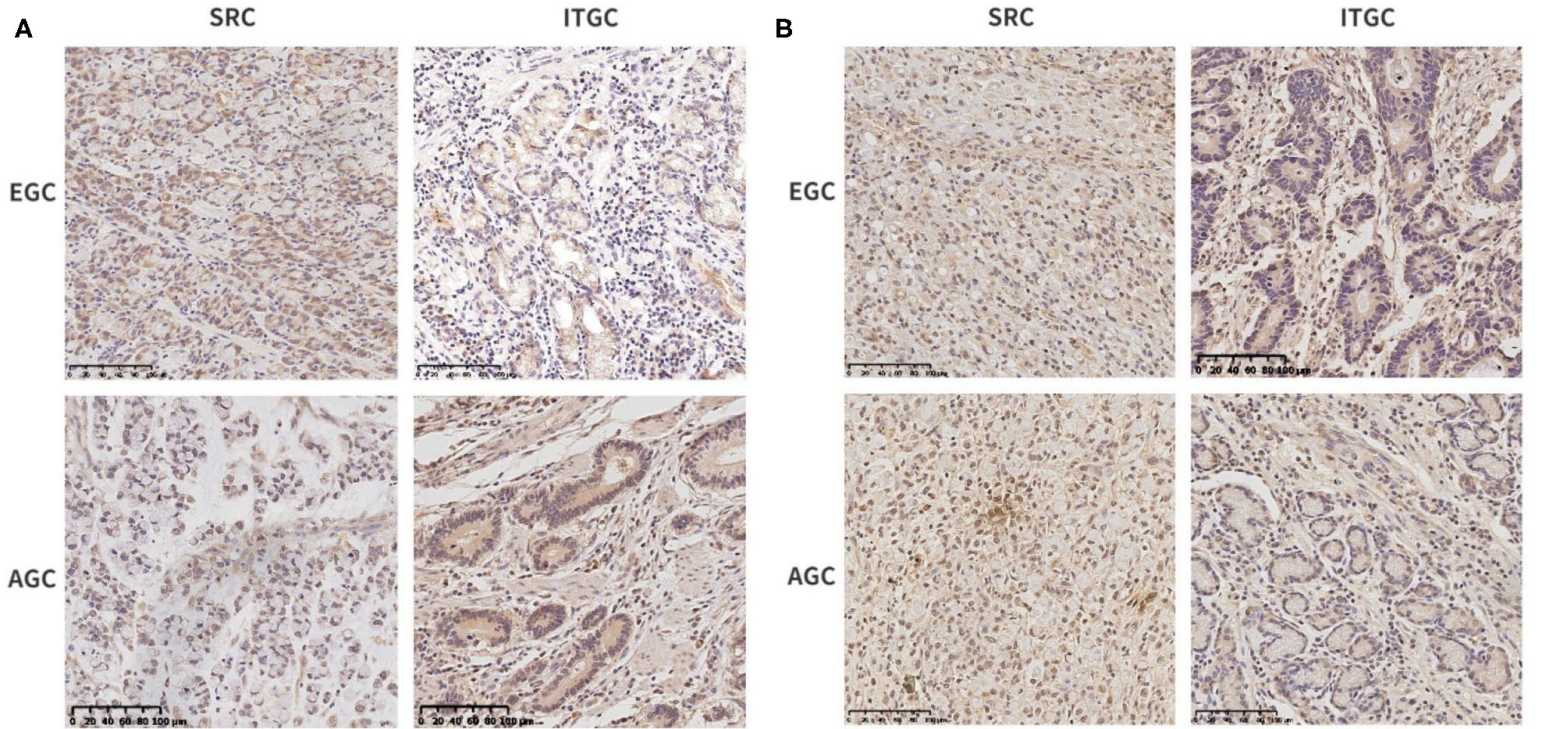
Immunohistochemical staining of SRC and intestinal-type gastric carcinoma (ITGC) samples for **(A)** THBS1 and **(B)** SERPINE1. The blue color stood for nuclear staining and the yellow color for target protein staining (scale bars: 100 μm).

For further confirmation of these genes at the protein level, we performed immunohistochemical staining in human samples (Figure 11). In EGC, there was no significant difference in the expression of THBS1 and ITGC in two types of gastric cancer, while in AGC, the expression of THBS1 and SERPINE1 in SRC was higher than that in ITGC (Table 5). In SRC, the expression of THBS1 and SERPINE1 was significantly higher in AGC than in EGC. In ITGC, the expression of THBS1 in AGC was significantly higher than that in EGC, while the expression of SERPINE1 in EGC was not significantly different from that in AGC (Table 6).

**Table 5:** Expression of THBS1 and SERPINE1 in signet-ring cell carcinoma and non-signet ring cell carcinoma.

	**SRC**		**ITGC**		***p***
	**+**	**–**	**+**	**–**	
**EGC**					
THBS1	5	26	4	19	0.902
SERPINE1	6	24	6	15	0.478
**AGC**					
THBS1	19	3	13	9	0.042
SERPINE1	17	5	10	12	0.030

*SRC, signet ring cell carcinoma; ITGC, intestinal-type gastric carcinoma; EGC, early gastric cancer; AGC, advanced gastric cancer*.

**Table 6:** Expression of THBS1 and SERPINE1 in early gastric cancer and advanced gastric cancer.

	**EGC**		**AGC**		***p***
	**+**	**–**	**+**	**–**	
**SRC**					
THBS1	5	26	19	3	<0.01
SERPINE1	6	24	17	5	<0.01
**ITGC**					
THBS1	4	19	13	9	<0.01
SERPINE1	6	15	10	12	0.252

*SRC, signet ring cell carcinoma; ITGC, intestinal-type gastric carcinoma; EGC, early gastric cancer; AGC, advanced gastric cancer*.

### Tumor Immune Infiltration Analysis

**Figure 12:**
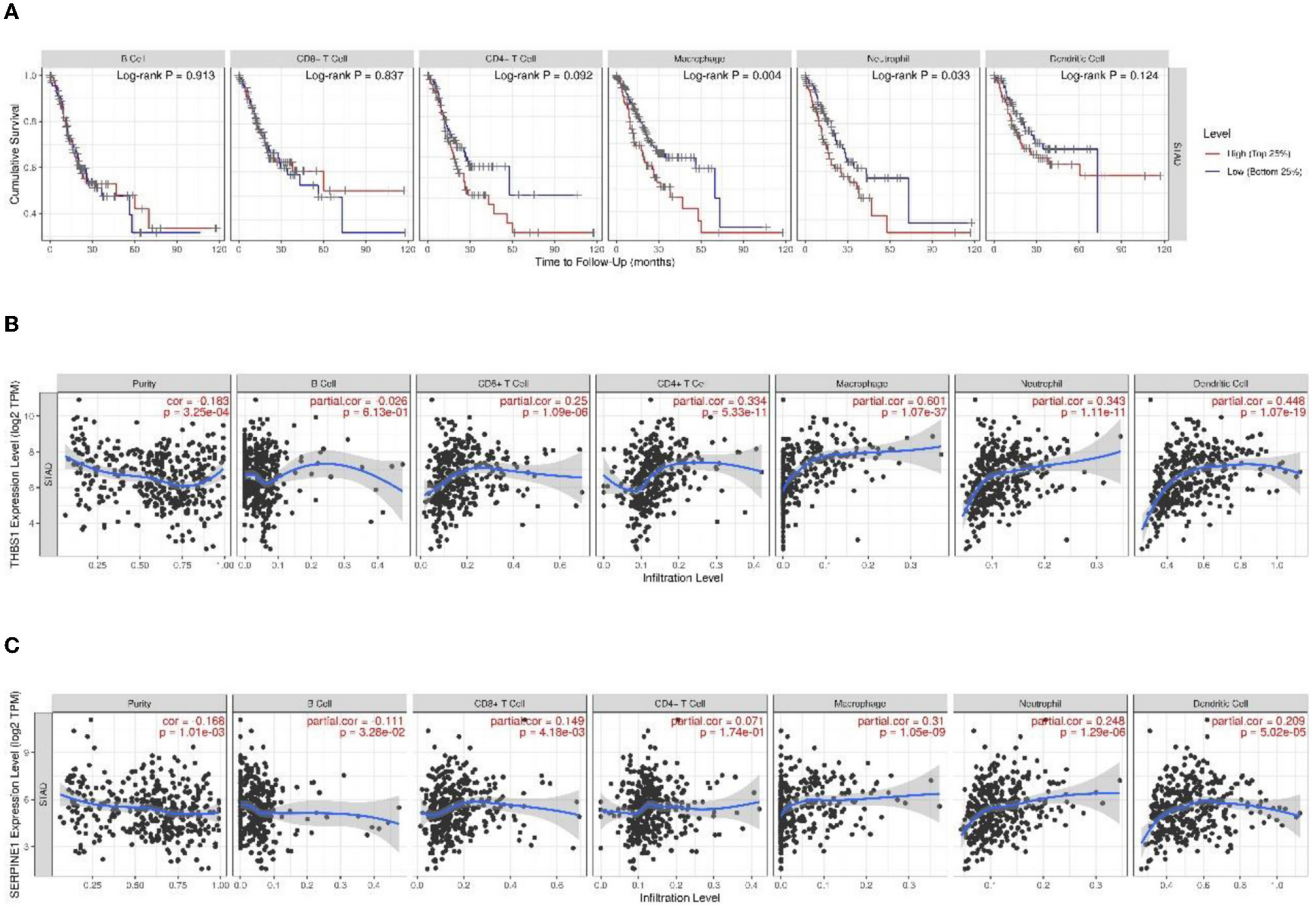
**(A)** Kaplan–Meier curves of gastric cancer stratified by immune cell abundance. Correlation with levels of immune infiltration (purity, B cells, CD8+ T cells, CD4+ T cells, macrophages, neutrophils, and dendritic cells) for **(B)** THBS1 and **(C)** SERPINE1. The correlation measurement is indicated by the partial correlation value using Spearman's partial rho and the statistical significance of the *p*-value.

The TIMER was used to explore the immunological microenvironment and identified correlations between levels of immune infiltration and expressions of the THBS1 and SERPINE1 in gastric cancer (Figure 12).

Survival analysis showed that macrophages (*p* = 0.004) and neutrophils (*p* = 0.033) were significantly associated with gastric cancer. THBS1 expression was significantly positively linked with immune infiltration of purity (*r* = −0.183, *p* < 0.001), CD8+ T cells (*r* = 0.25, *p* = 1.09 × 10^−6^), CD4+ T cells (*r* = 0.334, *p* = 5.33 × 10^−11^), macrophages (*r* = 0.601, *p* = 1.07 × 10^−37^), neutrophils (*r* = 0.343, *p* = 1.11 × 10^−11^), and DCs (*r* = 0.448, *p* = 1.07 × 10^−19^). The SERPINE1 expression was significantly positively linked with immune infiltration of purity (*r* = −0.168, *p* = 0.001), B cells (*r* = −0.111, *p* = 0.033), CD8+ T cells (*r* = 0.149, *p* = 0.004), macrophages (*r* = 0.310, *p* = 1.05 × 10^−9^), neutrophils (*r* = 0.248, *p* = 1.29 × 10^−6^), and DCs (*r* = 0.209, *p* = 5.02 × 10^−5^).

## Discussion

In recent years, a growing number of studies in Asian countries has shown that the prognosis of SRC depends on the pathological grading and staging, with better outcomes in SRC than in NSRC in EGC and a reversal in AGC (6–13). Previous studies using the SEER database did not show significant differences between SRC and NSRC (14, 15, 27). Gastric cancer is a mixture of various subtypes of tumors, and previous studies have shown that the clinical characteristics of different tumor types vary greatly (28–30). The cause of such results may be the heterogeneity of the tumor, which is induced by the different selection criteria. The currently recognized cause of intestinal-type gastric cancer is long-term chronic atrophic inflammation (31), the pathogenesis of other types of gastric cancer is unknown, and the clinicopathological characteristics and prognosis vary from one type to another. This study selected only ITGC and SRC, excluding mucinous adenocarcinoma, mixed adenocarcinoma, and other rare types.

Our results suggest that the clinical characteristics of SRC differ significantly from those of intestinal-type gastric adenocarcinoma. One difference is that SRC develops at an earlier age, approximately 7 years earlier than ITGC. The second difference lies in the sex distribution. Approximately half of patients with SRC are female, even though gastric cancer is generally considered to be a predominantly male cancer (32). Studies have shown that younger women have higher levels of estrogen receptors, so sex hormones may play a role in age and sex differences (33, 34). Other studies have shown that more than 80.0% of SRCs express estrogen receptors and are more likely to metastasize to the ovary, suggesting that SRCs have a higher affinity for estrogen (35). SRCs exhibit more middle and lower third tumor locations than the upper locations in the total population and are more likely to present with diffuse infiltrating gastric cancer in AGC. Some studies show that Mist1+ stem cells in the gastric isthmus can be transformed into SRCs in the absence of E-cadherin (36), which may be why SRCs are more frequently located in the middle third of the stomach. All of these findings reinforce the idea that SRC and ITGC may be two completely different diseases (37, 38).

Herein, we believe that stage adjustment is necessary to analyze the prognosis of SRC. SRC was associated with a better prognosis in early gastric cancer but a worse survival in advanced gastric cancer. These results may suggest that mutated genes controlling SRC progression may play a role in later stages of the disease. However, no studies have been conducted to elucidate how the gene level causes a difference in clinicopathological features between SRC and ITGC.

We identified 256 DEGs (119 upregulated and 137 downregulated) between SRC and ITGC in TCGA data, which may help us further explore the key reasons for the differential prognosis of the two types of gastric cancer. The genes THBS1, SERPINE1, VTN, and FGF2 were identified as genes of interest through functional enrichment analysis and PPI analysis. GO enrichment analysis showed that they were mainly related to biological processes such as wound healing, cell chemotaxis, and ECM tissue. These biological processes are at the core of our enrichment term network and may be closely related to the characteristics of SRC. Further survival analysis showed that THBS1, SERPINE1, and VTN were significantly associated with the prognosis of gastric cancer. Correlation analysis showed that THBS1, SERPINE, and FGF2 were correlated. In our study, it was found that the expression of THBS1 and SERPINE1 was significantly different in SRC and ITGC, as well as in EGC and AGC. It is reasonable to assume that THBS1 and SERPINE1 may have potentially important functions.

According to our study, SRC has more T4 and N3 distribution in pathological stages than ITGC; this may be the reason why SRC shows more malignancy in AGC than ITGC. Thrombospondin1 is an extracellular glycoprotein that has been shown to play a role in cell invasion and migration (39). Some studies have confirmed that THBS-1 protein is mainly located in myofibroblasts of the tumor stroma and is significantly associated with lymph node metastasis of gastric cancer (40). It has also been proven that FGF7/FGFR2 signaling promotes the invasion and migration of gastric cancer by upregulating THBS1 (41). Serpin peptidase inhibitor, clade E, member 1 can prevent excessive proteolysis and maintain the integrity of the ECM, which is necessary for capillary morphogenesis, cell migration, and tumor invasion (42). Studies have shown that the lncRNA NKX2-1-AS1 can activate the VEGFR-2 signaling pathway through SERPINE1 to promote tumor progression and angiogenesis in gastric cancer (43). Tumor cell line validation also showed higher expression of THBS1 and SERPINE1 in metastatic cancer cell lines. Given the role of THBS1 and SERPINE1 in tumor invasion and metastasis, it may explain the higher degree of malignancy in advanced SRC to some extent.

We also performed a correlation analysis of tumor-infiltrating immune cells. In gastric cancer, macrophages and neutrophils are significantly associated with prognosis. THBS1 and SERPINE1 were associated with multiple immune cell infiltrates, with the correlation between THBS1 and macrophages up to 0.601 (*p* = 1.07 × 10^−37^). Tumor-associated macrophages (TAM) are important components of tumor microenvironment and regulate tumor progression. TAM can secrete matrix metalloproteinase (MMP), serine protease and cathepsin to mediate ECM degradation and cell–ECM interaction to promote tumor cell invasion and migration (44, 45).

There are some limitations in our study that must be considered. First, although the use of a large database can reduce the bias due to differences in patient distribution to some extent, these data also limited our study because perioperative chemotherapy, which is critical to prognosis, was missing. The surgery type and the extent of lymph node dissection (D1, D2) were not recorded in patients who underwent surgical resection. Therefore, more cohort studies should be conducted.

Second, two parts of this study were obtained from the SEER database and TCGA database, and both are maintained by the National Cancer Institute. Although the inclusion criteria for the two parts of this study were basically the same, due to the defects of the database itself, there was a huge difference in the proportion of SRC and ITGC cases. Thus, it is not appropriate for us to add other features to the grouping. These deficiencies may have partially influenced the results, as evidenced by the fact that CDH1 (46) and CDS1 expression did not differ between two groups. Moreover, since the number of SRCs in the TCGA database is too small, it is difficult to conduct grouping for subsequent analysis of genes of interest. Although our results were validated by immunohistochemistry, more studies on the single-cell sequencing of SRC are needed. Further mechanistic validation for the genes of interest will be further implemented.

## Conclusions

There were significant differences in the clinicopathological features and prognosis between SRC and ITGC. These results suggest that SRC and ITGC may be two distinct types of tumors with different pathogeneses. We found many codifferentially expressed genes and important pathways between SRC and ITGC. THBS1 and SERPINE1 were significantly differentially expressed in the two types of gastric cancer, and may have potentially important functions.

## Data Availability Statement

The original contributions presented in the study are included in the article/supplementary material, further inquiries can be directed to the corresponding author.

## Ethics Statement

Ethical review and approval was not required for the study on human participants in accordance with the local legislation and institutional requirements. Written informed consent for participation was not required for this study in accordance with the national legislation and the institutional requirements.

## Author Contributions

JM analyses the data and statistics and drafted the manuscripts. YM was responsible for literature search, manuscript preparation, and contributed to some of the pictures. WF, LG, and XZ responsible for the design of the study, reviewed the manuscript, provided feedback, and provided financial support. All authors contributed to the article and approved the submitted version.

## Funding

This work was supported by grants from the National Natural Science Foundation of China (No. 82003153), Beijing Municipal Science and Technology Commission (No. Z131100004013036), and National Multidisciplinary Cooperative Diagnosis and Treatment Capacity Building Project for Major Diseases: Comprehensive Diagnosis and Treatment of gastrointestinal Tumors (No. A63445-24).

## Conflict of Interest

The authors declare that the research was conducted in the absence of any commercial or financial relationships that could be construed as a potential conflict of interest.

## Publisher's Note

All claims expressed in this article are solely those of the authors and do not necessarily represent those of their affiliated organizations, or those of the publisher, the editors and the reviewers. Any product that may be evaluated in this article, or claim that may be made by its manufacturer, is not guaranteed or endorsed by the publisher.
